# Effects of grain-based diets on the rumen and fecal bacterial communities of the North American bison (*Bison bison*)

**DOI:** 10.3389/fmicb.2023.1163423

**Published:** 2023-07-06

**Authors:** Anlly Fresno Rueda, Jason Eric Griffith, Carter Kruse, Benoit St-Pierre

**Affiliations:** ^1^Department of Animal Science, South Dakota State University, Brookings, SD, United States; ^2^Turner Institute of Ecoagriculture, Bozeman, MT, United States

**Keywords:** 16S rRNA gene, bison, rumen, feces, diet, bacterial composition, microbiome

## Abstract

To overcome the challenges of pasture-finishing of bison, producers commonly feed them with higher energy, grain-based diets to reach the desired market weight. However, decades of research on domesticated ruminants have shown that such diets can have profound effects on the composition of gut microbial communities. To gain further insight, the 16S rRNA gene-based study described in this report aimed to compare the composition of ruminal and fecal bacterial communities from two herds of bison heifers (*n* = 20/herd) raised on different ranches that were both transitioned from native pasture to a grain-based, free-choice diet for ~100 days prior to slaughter. Comparative analyses of operational taxonomic unit (OTU) composition, either by alpha diversity indices, principal coordinate analysis (PCoA), or on the most abundant individual OTUs, showed the dramatic effect of a diet on the composition of both rumen and fecal bacterial communities in bison. Indeed, feeding a grain-based diet resulted in a lower number of rumen and fecal bacterial OTUs, respectively, compared to grazing on pasture (*p* < 0.05). PCoA revealed that the composition of the rumen and fecal bacterial communities from the two herds was more similar when they were grazing on native pastures compared to when they were fed a grain-based, free-choice diet. Finally, a comparative analysis of the 20 most abundant OTUs from the rumen and fecal communities further showed that the representation of all these species-level bacterial groups differed (*p* < 0.05) between the two dietary treatments. Together, these results provide further insights into the rumen and fecal microbiomes of grazing bison and their response to grain-based diet regimens commonly used in intensive ruminant production systems.

## Introduction

For thousands of years, bison were the predominant land mammal in North America, with tens of millions of them roaming a wide range of habitats from Alaska to Mexico (Lott, 2002; Heintzman et al., 2016; Martin et al., 2022). After narrowly escaping extinction nearly a century ago, bison populations have recovered owing to the combined efforts of conservation groups and producers (Isenberg, 2020). Since then, their populations have continued to increase, with ~360,000 plain bison in North America [NBA (National Bison Association), 2022], including 30,000 in public herds (Gates et al., 2010) and 20,000 in tribal sector herds (ITBC, 2021). In recent years, bison production has greatly contributed to restoring populations as it has grown into a niche industry serving an increasing number of consumers looking for alternative red meat options. While bison have evolved as grazing animals, pasture-finishing of bison remains a challenge because of the time and space required to bring animals to market (Carter et al., 2010). To overcome this issue, many bison producers follow the commonly used strategy in the ruminant livestock industry of finishing animals by feeding diets that include grain or concentrate (Huntington, 1997; Huntington et al., 2006). As these diets are more easily digestible and have a higher energy content, they improve not only animal performance but also overall production efficiency in ruminants (Gómez et al., 2016) and, in the case of bison, can also help to mitigate reduced winter intake and other seasonal effects. While this strategy allows producers to expedite weight gain to reach the desired market weight, decades of research on cattle and other ruminant livestock have shown that a diet based on easily fermentable carbohydrates can have adverse physiological effects if administered in excess. Most notably, these effects include changing the composition of gut microorganisms, negatively impacting the gut environment, and affecting the physiology of both the foregut and the hindgut segments, which can compromise the health of the host animal.

Bison, similar to other ruminants, rely on the metabolic activities of gut symbiotic microorganisms to break down and ferment plant structural polysaccharides into nutrients they can absorb and assimilate (Macfarlane and Macfarlane, 1993). In ruminants, fermentation of feedstocks takes place in the rumen, the largest segment of their compartmentalized stomach, which is consequently the main habitat for microbial symbionts in these herbivores (Hungate, 1966; Hofmann, 1989; Dehority, 2003). Rumen microorganisms include bacterial, archaeal, protozoal, and fungal species, and these species assemble into complex microbial ecosystems that develop from trophic relationships and intricate functional networks that form among microorganisms (Johnson et al., 2012). Rumen microbial communities work synergistically to ferment ingested feedstocks, producing short-chain fatty acids (SCFAs) and microbial proteins that are used by their host as sources of energy and amino acids, respectively (Lengowski et al., 2016; Wang et al., 2017). Since each ruminal microbial species is a metabolic specialist, i.e., each type of microorganism is limited in the number and types of metabolic functions it can perform, membership in a community overcomes individual limitations by sharing benefits from the complementary metabolic capabilities of other community members (Wallace, 2008). In other words, individual microbial species can maximize their efficiency because they can depend on other members of their community to provide them with nutrients they are unable to synthesize. In the context of the nutrient complexity of a ruminant diet, the ability of the rumen to metabolize the different components of feed is the result of the complementary metabolic activities of a wide array of microbial specialists (Wallace, 2008). The greater complexity of ruminal microbial communities is a characteristic of grass- or forage-based diets compared to grain- or concentrate-based diets, largely because of the higher diversity of substrates in the former (Henderson et al., 2015).

It has been estimated that 95% of ruminal bacterial species have yet to be characterized in domesticated ruminant livestock species, highlighting that the function and metabolic potential of most rumen microorganisms remain largely unexplored (Creevey et al., 2014). Our current knowledge gap on bison gut microbial communities is even more pronounced than for domesticated ruminants, with comparatively few published studies (Towne et al., 1988; Oss et al., 2016; Bergmann, 2017; Griffith et al., 2017; Ribeiro et al., 2017; Rico et al., 2021). As the effects of grain-based diets on the bison gastrointestinal environment remain largely unexplored, the main objective of this study was to compare the diversity and composition of the rumen and fecal bacterial communities of bison that were transitioned from grazing on native pastures to a grain-based free-choice diet. An additional benefit of this analysis was that it provided further insights into the largely unexplored gut microbiomes of a highly successful North American ruminant.

## Materials and methods

### Animals and sample collection

The bison sampled in this study were raised at two different locations: Standing Butte Ranch (South Dakota, USA; 44.5824807°N 100.8526463°W) and Blue Creek Ranch (Nebraska, USA; 41.6042°N 102.3386°W). The animals under study were managed under standard practices used by both ranches to finish bison for market. At each ranch, 20 heifers (25–26 months of age), which prior to this study had only foraged on native grasses and forbs (Supplementary Table 1), were transitioned to a limited free-choice, grain-based diet for a period of 97–101 days, with *ad libitum* access to corn, alfalfa hay, and grass hay. During this time, the 20 heifers at each ranch were maintained in loose confinement in a single pen, with ample room to run and move freely (74.3–92.9 m^2^/animal). While daily intake was not measured individually, it averaged 6.8 kg of corn, 4.5 kg of alfalfa hay, and 1.8 kg of grass hay.

Procedures for sample collections were approved by the South Dakota State University Institutional Animal Care and Use Committee. Rumen and fecal samples were collected from the same heifers at two different time points: (1) at the end of their time on pasture, just prior to their transition to the free-choice, grain-based diet, and (2) at the end of the free-choice diet period (i.e., 97–101 days after the diet transition). During the sample collection process, the heifers were individually secured in a squeeze chute. Rumen fluid was obtained using a stomach tube. To prevent contamination from saliva, the initial 25–50 ml of the collected fluid was discarded. Moreover, ~200 ml of rumen fluid was then collected, of which ~30 ml were frozen and stored for subsequent analysis. Fecal samples (~30 ml/heifer) were collected using the rectal grab method. These samples were subsequently frozen and stored for later analysis. We could not obtain both types of samples for all the animals on trial. Out of a possible total of 80 samples of each type, 76 rumen fluid samples were collected [Standing Butte grass (SBGrass) = 19; Standing Butte grain-based/free-choice diet (SBGrain) = 18; Blue Creek grass (BCGrass) = 20; Blue Creek grain-based/free-choice diet (BCGrain) = 19], while 71 fecal samples were collected (SBGrass = 18; SBGrain = 17; BCGrass = 19; BCGrain = 17).

### Microbial DNA extraction and PCR amplification of the 16S rRNA gene

Microbial genomic DNA was extracted from the individual rumen (*n* = 76) and fecal (*n* = 71) samples using a bead-beating plus column approach as described in a previous study (Yu and Morrison, 2004), which included using the QIAamp DNA Mini Kit (Qiagen, Hilden, Germany). The V1–V3 regions of the bacterial 16S rRNA gene were targeted by PCR using the universal forward 27F-5′AGAGTTTGATCMTGCTCAG (Edwards et al., 1989) and reverse 519R-5′GWATTACCGCGCGCGCTG (Lane et al., 1985) primers. PCR was performed under the following conditions: an initial denaturing step at 98^o^C (4 min), followed by 35 cycles of denaturation at 98^o^C (10 s), annealing at 50^o^C (30 s), and an extension at 72^o^C (30 s). Amplification ended with an extension period of 10 min at 72^o^C. Agarose gel electrophoresis was then performed to confirm the quality and molecular weight (~500 bp) of the PCR-amplified DNA, which was recovered using the QiaexII Gel extraction kit (Qiagen, Hilden, Germany). Purified PCR samples (at least 400 ng) were submitted to Molecular Research DNA (MRDNA, Shallowater, TX, USA) for sequencing with the Illumina MiSeq 2X300 platform to generate overlapping paired-end reads.

### Bacterial composition analyses

Sequence data were processed using a combination of custom-written Perl scripts (Bandarupalli and St-Pierre, 2020) and publicly available software. Sequences from merged, overlapping paired-end reads corresponding to V1–V3 amplicons generated from the 16S rRNA bacterial gene were first screened to meet the following criteria: the presence of both intact 27F and 519R primer sequences, a minimal average Phred quality score of Q33, and a length between 400 and 580 nt. After quality filtering, amplicon sequences were aligned and clustered into operational taxonomic units (OTUs) using a sequence dissimilarity cutoff of 4%. Based on the reports by Kim et al. (2011) and Johnson et al. (2019), this threshold is more suitable for the V1–V3 region than the 3% cutoff that is typically used indiscriminately for the clustering of 16S rRNA sequence data, regardless of the variable regions targeted for analysis. Following OTU clustering, three independent approaches were used to identify artifacts. First, OTUs were screened for chimeric sequences using the “chimera.slayer” (Haas et al., 2011) and “chimera.uchime” (Edgar et al., 2011) commands from the MOTHUR (v.1.36.1) open-source software package (Schloss et al., 2009). Moreover, the 5′ and 3′ ends of OTUs were evaluated using a database alignment search-based approach; when compared to their closest match of equal or longer sequence length from the NCBI “nt” database, as determined using BLAST (Altschul et al., 1997), OTUs with more than five nucleotides missing from the 5′ or 3′ ends of their respective alignments were designated as artifacts. Finally, OTUs with only one or two assigned reads were subjected to an additional screen, where only sequences with a perfect or near-perfect match (maximum 1% of dissimilar nucleotides) to a sequence in the NCBI “nt” database were kept for analysis. All OTUs and their assigned reads that were flagged during these screens were subsequently removed from further analyses. The resulting curated OTUs were then analyzed for taxonomic assignment using two strategies. For all OTUs, the RDP Classifier determined phylum and family-level affiliations (Wang et al., 2007). The closest valid relatives for the most abundant OTUs were identified by searches with BLAST against the “refseq_rna” database (Altschul et al., 1997).

Using the MOTHUR (v.1.36.1) open-source software package (Schloss et al., 2009), the alpha diversity indices “Observed OTUs,” “Chao,” “Ace” and “Shannon” were determined using the “summary.single” command. For beta diversity analysis in MOTHUR (v.1.36.1), Bray–Curtis distances were first calculated using “summary.shared,” followed by “pcoa” for principal coordinate analysis (PCoA). Curated datasets were rarefied to 3,500 sequences using custom Perl scripts to perform alpha and beta diversity analyses. Plots for alpha diversity and PCoA were generated using the Tableau Visualization Software (Version 2020.4, https://www.tableau.com/products/new-features).

### Statistical analyses

All statistical analyses were performed using the RStudio Statistical Software (Version 1.3.959 © 2009-2020 RStudio, PBC). To determine whether parametric or non-parametric statistical tests should be used, the Shapiro–Wilk test was conducted to verify the normality assumption. An analysis of variance (ANOVA), with a *post-hoc* “HSD.test” pair-wise function was used to compare alpha diversity indices. For statistical testing of taxonomic groups and individual OTUs, the Kruskal–Wallis sum-rank test was performed among experimental groups, concomitant with the pairwise Wilcoxon sum-rank test, which included the Benjamini–Hochberg correction for controlling the false discovery rate, to compare abundances between sample group pairs. The “Adonis” function from the vegan package (Oksanen et al., 2013) was used for permutational multivariate analysis (PERMANOVA, 999 permutations) to detect statistical differences among sample sets, followed by the “pair-wise.adonis” function from the “devtools” package to identify pairs of sample groups that were different. For all analyses, a *p*-value of ≤ 0.05 was considered significant.

## Results

### Taxonomic profiles of rumen bacterial communities

**Figure 1:**
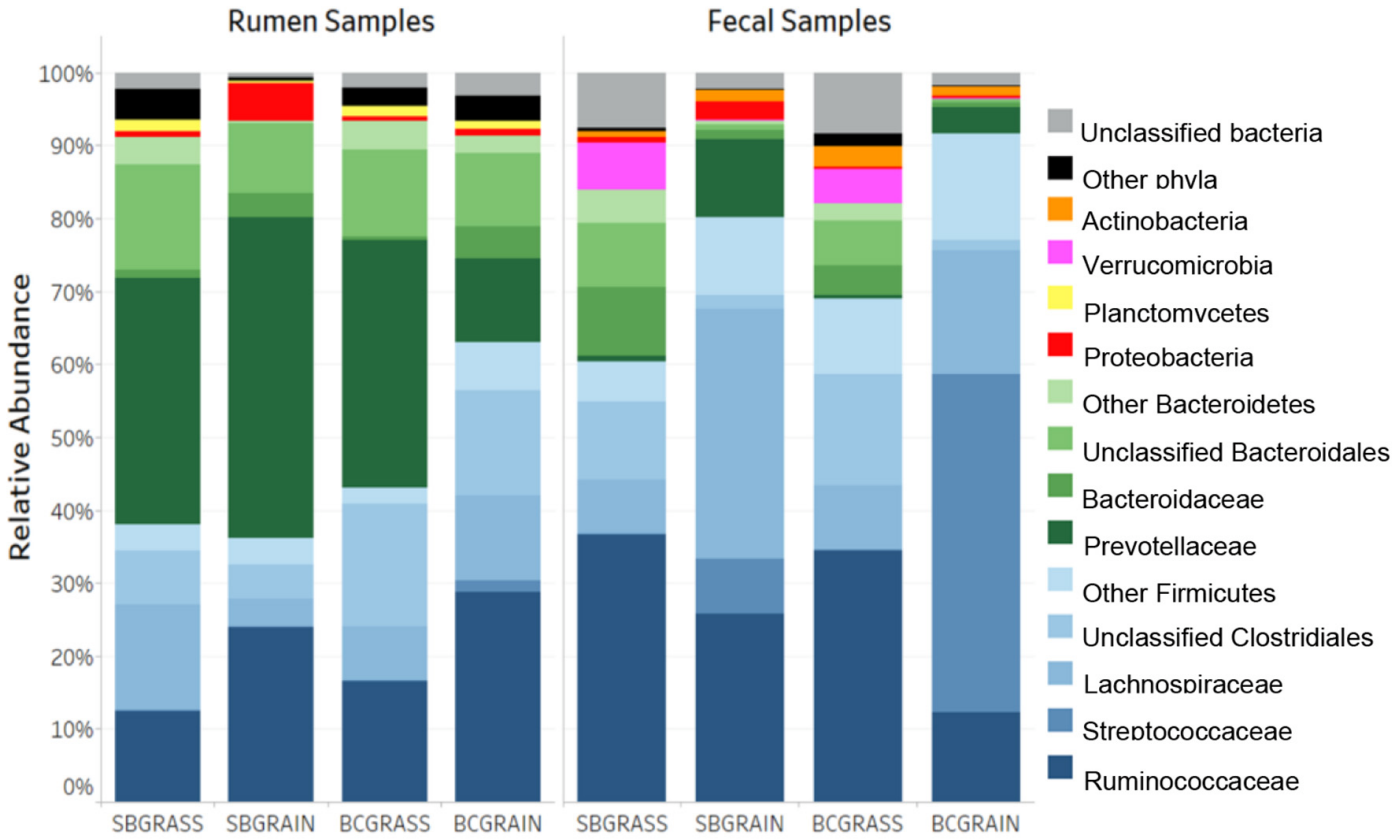
Phylum- and family-level taxonomic composition of the rumen and fecal bacterial communities from heifers at two ranches that transitioned from pasture to a free-choice grain diet. SBGrass, Standing Butte grass; SBGrain, Standing Butte grain; BCGrass, Blue Creek grass; BCGrain, Blue Creek grain.

A total of 662,651 (μ = 8,719 ± 4,837/sample) full-length, quality-filtered sequences were obtained from V1–V3 amplicons generated from the 16S rRNA gene for rumen samples collected from bison heifers raised at two different ranches on two distinct diet regimens. Bacteroidetes were the most highly represented phylum among three of the four rumen sample groups analyzed, with BCGrain heifers showing the lowest abundance (*p* < 0.05; Figure 1, Table 1). A similar pattern was also observed for Prevotellaceae (*p* < 0.05), which were by far the most abundant and well-characterized Bacteroidetes family in bison rumen (Table 1).

**Table 1:** Major taxonomic groups identified in the rumen of heifers from two ranches that transitioned from pasture to a free-choice grain diet.

**Taxa**	**Relative abundance (%)**				***p*-value^#^**
	**SBGrass**	**SBGrain**	**BCGrass**	**BCGrain**	
**Firmicutes** ^**#**^	**38.01** ^**a**^	**36.14** ^**a**^	**43.02** ^**a**^	**63.03** ^**b**^	< 0.001
Ruminococcaceae^#^	12.52^a^	23.91^abc^	16.72^b^	28.72^c^	< 0.001
Streptococcaceae^#^	0.09^a^	0.21^b^	0.01^c^	1.55^d^	< 0.001
Lachnospiraceae^#^	14.35^a^	3.72^b^	7.28^c^	11.72^a^	< 0.001
Unclassified Clostridiales^$^	7.44	4.67	16.92	14.51	–
Other Firmicutes^$^	3.61	3.63	2.10	6.53	–
**Bacteroidetes** ^**#**^	**53.12** ^**a**^	**57.27** ^**a**^	**50.39** ^**a**^	**28.30** ^**b**^	< 0.001
Prevotellaceae^#^	33.83^a^	44.01^a^	33.94^a^	11.49^b^	< 0.001
Bacteroidaceae	1.10	3.32	0.53	4.45	0.0723
Unclassified Bacteroidales^$^	14.47	9.62	12.00	9.99	–
Other Bacteroidetes^$^	3.71	0.32	3.92	2.37	–
**Proteobacteria**	**0.84** ^**a**^	**5.08** ^**a**^	**0.65** ^**a**^	**0.89** ^**a**^	0.6217
Succinivibrionaceae	0.08^a^	2.50^b^	0.04^a^	0.24^a^	< 0.001
**Planctomycetes** ^**#**^	**1.60** ^**a**^	**0.37** ^**b**^	**1.40** ^**a**^	**1.18** ^**a**^	< 0.001
**Other phyla** ^**$**^	**4.18**	**0.51**	**2.39**	**3.46**	–
**Unclassified bacteria** ^**$**^	**2.25**	**0.62**	**2.15**	**3.15**	–

SBGrass, Standing Butte grass; SBGrain, Standing Butte grain; BCGrass, Blue Creek grass; BCGrain, Blue Creek grain.

^#^Taxa showing a statistically significant difference by the Kruskal–Wallis sum rank test (p < 0.05).

Different superscripts in the same row indicate that the groups were significantly different by the Wilcoxon pairwise test (p < 0.05).

^$^Statistical test not performed because of group heterogeneity. Phylum-level groups are highlighted in bold.

Firmicutes were also very well represented in the rumen of the sampled heifers, with the highest levels observed in individuals from the BCGrain group (*p* < 0.05; Figure 1, Table 1). Among Firmicutes, Ruminococcaceae were found to be in higher abundance in both BCGrain and SBGrain, but statistical support for these differences was only observed for the heifers raised at the Blue Creek ranch (Table 1). Lachnospiraceae, which were the second most abundant Firmicutes family identified in rumen samples, displayed a more complex pattern of abundance, with the highest levels observed in SBGrass (*p* < 0.05), in contrast to the highest levels found in BCGrain heifers.

The phylum Planctomycetes was observed in rumen samples from both locations and diets, with abundances ranging between 0.37 and 1.60% across all sample groups (Figure 1, Table 1), but the levels of rumen Planctomycetes were found to be the lowest (*p* < 0.05) in the SBGrain heifers. Numerically, Proteobacteria appeared to be in the highest abundance in the same set of samples, mostly as a result of three samples from this group that had elevated levels of sequences from this phylum (8.9%, 27.6%, and 41.0%—Supplementary Figure 1). In these samples, Succinivibrionaceae represented by far the most abundant Proteobacteria family (Table 1).

### Taxonomic profiles of fecal bacterial communities

Because they can be collected using minimally invasive methods, fecal samples are commonly used as a proxy for investigating bacterial communities in the hindgut. A total of 762,870 (μ = 10,744 ± 4,813/sample) full-length, quality-filtered 16S rRNA (V1–V3) sequences were analyzed from fecal samples collected from the same group of bison heifers. Firmicutes were found to be more abundant in fecal bacterial communities compared to those of the rumen. In both locations, the highest representation of this phylum among fecal samples was observed in bison fed a grain-based diet (*p* < 0.05; Figure 1, Table 2). In contrast to the rumen samples, Ruminococcaceae were more abundant in fecal samples from grass-fed bison, while Lachnospiraceae showed an opposite abundance pattern (*p* < 0.05; Table 2). Streptococcaceae were found to have a much higher representation in fecal communities from grain-based diets (*p* < 0.05), with 94.1X and 290.1X greater levels than in grass-based samples for the Standing Butte and Blue Creek ranches, respectively.

**Table 2:** Major taxonomic groups identified in the feces of heifers from two ranches that transitioned from pasture to a free-choice grain diet.

**Taxa**	**Mean relative abundance (%)**				***p*-value^#^**
	**SBGrass**	**SBGrain**	**BCGrass**	**BCGrain**	
**Firmicutes** ^**#**^	**60.33** ^**a**^	**80.18** ^**b**^	**68.96** ^**c**^	**91.59** ^**d**^	< 0.001
Ruminococcaceae^#^	36.74^a^	25.74^b^	34.41^a^	12.24^c^	< 0.001
Streptococcaceae^#^	0.08^a^	7.53^b^	0.16^c^	46.45^d^	< 0.001
Lachnospiraceae^#^	7.38^a^	34.29^b^	8.79^c^	16.86^d^	< 0.001
Unclassified Clostridiales^#^	10.63^a^	1.89^b^	15.25^c^	1.43^b^	< 0.001
Other Firmicutes	5.51	10.73	10.36	14.61	–
**Bacteroidetes** ^**#**^	**23.67** ^**a**^	**13.39** ^**b**^	**13.11** ^**b**^	**4.94** ^**c**^	< 0.001
Prevotellaceae^#^	0.83^a^	10.75^b^	0.52^c^	3.67^d^	< 0.001
Bacteroidaceae^#^	9.42^a^	1.16^b^	4.05^c^	0.65^b^	< 0.001
Unclassified Bacteroidales	8.84^a^	0.78^b^	6.20^c^	0.38^d^	< 0.001
Other Bacteroidetes	4.58	0.70	2.35	0.24	–
**Proteobacteria** ^**#**^	**0.76** ^**a**^	**2.49** ^**b**^	**0.40** ^**c**^	**0.27** ^**d**^	< 0.001
Succinivibrionaceae^#^	< 0.01^a^	1.93^b^	0.01^a^	0.18^c^	< 0.001
**Verrucomicrobia** ^**#**^	**6.38** ^**a**^	**< 0.01** ^**b**^	**4.63** ^**a**^	**< 0.01** ^**b**^	< 0.001
Akkermansiaceae^#^	6.31^a^	< 0.01^b^	4.60^a^	< 0.01^b^	< 0.001
**Actinobacteria** ^**#**^	**0.86** ^**a**^	**1.68** ^**b**^	**2.77** ^**c**^	**1.36a** ^**b**^	< 0.001
Atopobiaceae^#^	0.34^a^	1.11^b^	0.99^b^	0.37^a^	< 0.001
**Other phyla**	**0.42**	**0.08**	**1.70**	**0.13**	–
**Unclassified bacteria**	**7.58**	**2.18**	**8.43**	**1.70**	–

SBGrass, Standing Butte grass; SBGrain, Standing Butte grain; BCGrass, Blue Creek grass; BCGrain, Blue Creek Grain.

^#^Taxa showing a statistically significant difference by the Kruskal–Wallis sum rank test (p < 0.05).

Different superscripts in the same row indicate that groups were significantly different by the Wilcoxon pairwise test.

^$^Statistical test not performed because of group heterogeneity. Phylum-level groups are highlighted in bold.

In contrast to rumen bacterial communities, Bacteroidetes were found in much lower numbers in fecal samples. Notably, Prevotellaceae and Bacteroidaceae, the two main families of Bacteroidetes identified in this study, showed an opposite pattern of abundance in fecal samples, with the former found at higher levels in heifers fed a grain-based diet, while the latter were more highly represented in grass-fed bison (*p* < 0.05; Table 2). Another difference observed between fecal and rumen bacterial communities was the prevalence of other minor phyla. Indeed, Verrucomicrobia and Actinobacteria were found in the third and fourth highest representations instead of Proteobacteria and Planctomycetes. Akkermansiaceae represented the most abundant family of Verrucomicrobia, with the highest levels observed in grass-fed bison (Table 2), while intermediate abundances within the same range were observed in fecal bacterial communities from heifers fed a grain-based diet. The taxonomic profile of individual fecal samples can be found in Supplementary Figure 2.

### Alpha and beta diversity analyses of the rumen and fecal bacterial communities

**Figure 2:**
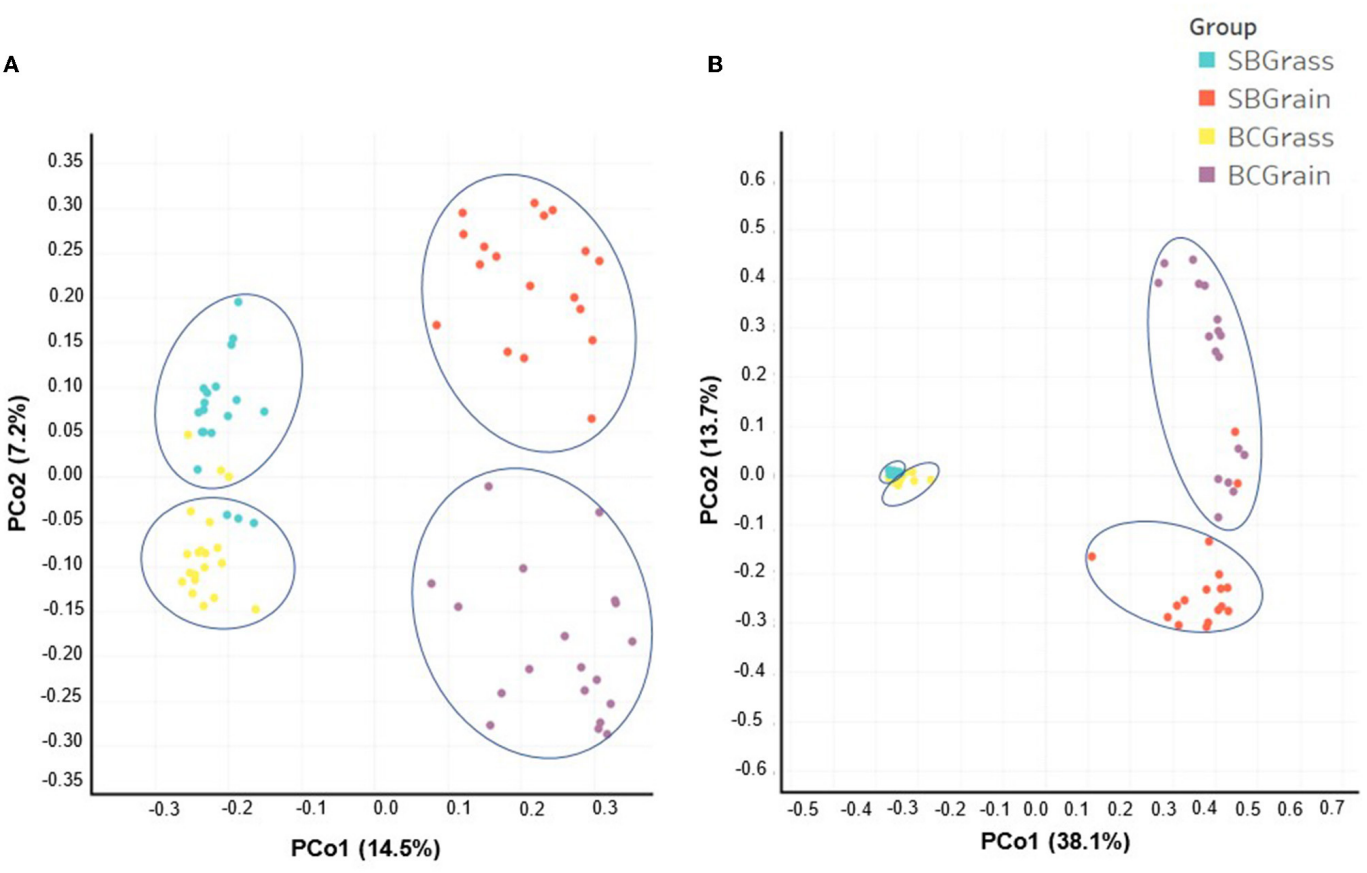
Comparison by principal coordinate analysis (PCoA) of the rumen **(A)** and fecal **(B)** bacterial communities from heifers at two ranches that transitioned from pasture to a free-choice grain diet. The *x*- and *y*-axes correspond to principal component 1 (PCo1) and principal component 2 (PCo2). Panel **(A)** (rumen samples): (SBGrass *n* = 19; SBGrain *n* = 18; BCGrass *n* = 20; BCGrain *n* = 19). Panel **(B)** (fecal samples): (SBGrass = 18; SBGrain = 17; BCGrass = 19; BCGrain = 17). Ellipses represent differences between experimental groups resolved by the PERMANOVA and Adonis tests (*p* = 0.001).

Indices of bacterial diversity based on operational taxonomic units (OTUs) showed statistical differences across each set of the rumen and fecal sample groups (Table 3). Overall, the rumen of bison heifers showed greater bacterial diversity (observed OTUs, Chao, and Ace) compared to fecal bacterial communities, and higher diversity was observed in samples from grass-fed bison in comparison to those fed grain-based diets (*p* < 0.05). PCoA further highlighted differences in composition, as it revealed four distinct clusters among each respective set of the rumen (Figure 2A) and fecal samples (Figure 2B). Notably, the dissimilarity between grass- and grain-based diets for both rumen (*p* < 0.001) and fecal (*p* < 0.001) communities was supported by PERMANOVA. The results from the PCoA also indicated that the microbial composition of the rumen and fecal bacterial communities from the two herds was more similar when on native grasses compared to the grain-based diet.

**Table 3:** Alpha-diversity indices from the rumen and fecal microbial communities of bison heifers that transitioned from pasture to free-choice grain-based diets.

**Index**	**SBGrass**	**SBGrain**	**BCGrass**	**BCGrain**	***p*-value**
**Rumen samples**					
Observed OTUs	1,920 ± 53.4^a^	886 ± 67.1^b^	2,165 ± 33.12^c^	812 ± 52.6^b^	< 0.001
Chao	7,239 ± 364^a^	3,325 ± 393.7^b^	10,416 ± 372.7^c^	1,418 ± 89.5^d^	< 0.001
Ace	15,105 ± 960.8^a^	6,624 ± 956.1^b^	23,694 ± 1111.6^c^	2,098 ± 150.7^d^	< 0.001
Shannon	7.01 ± 0.06^a^	4.73 ± 0.21^b^	7.18 ± 0.04^a^	5.26 ± 0.18^c^	< 0.001
Simpson	0.00 ± 0.00^a^	0.07 ± 0.01^b^	0.00 ± 0.00^a^	0.03 ± 0.01^c^	< 0.001
**Fecal samples**					
Observed OTUs	1,546 ± 32.6^a^	476 ± 35.7^b^	1,286 ± 30.1^c^	282 ± 16.9^d^	< 0.001
Chao	3,176 ± 155.5^a^	901 ± 66.9^b^	2,024 ± 70.7^c^	470 ± 28.4^d^	< 0.001
Ace	5,055 ± 383.1^a^	1,433 ± 115.9^b^	2,496 ± 152.9^c^	699 ± 55.6^b^	< 0.001
Shannon	6.61 ± 0.05^a^	4.31 ± 0.17^b^	6.53 ± 0.04^a^	3.07 ± 0.18^c^	< 0.001
Simpson	0.01 ± 0.00^a^	0.06 ± 0.01^a^	0.00 ± 0.00^a^	0.22 ± 0.03^b^	< 0.001

SBGrass, Standing Butte grass; SBGrain, Standing Butte grain; BCGrass, Blue Creek grass; BCGrain, Blue Creek grain.

Values are presented as means.

Different superscripts in the same row indicate that groups were significantly different; pair-wise differences between alpha diversity indices were calculated using the Tukey HSD.test, with the significance level set to a p-value of < 0.05.

### OTU composition analysis of rumen bacterial communities

A total of 85,602 unique OTUs were identified across all rumen samples in this study (Supplementary Table 2), of which 0.6% of OTUs were predicted to correspond to known or valid bacterial species. In light of the differences in bacterial composition that were revealed by PCoA, further analyses were conducted on the 20 most abundant rumen OTUs, which were found to differ in abundance between grass- and grain-based diets at both locations (*p* < 0.05; Table 4). From the samples collected at the Standing Butte ranch, 14 rumen OTUs were in higher abundance in the SBGrain group, including Bb-00002, Bb-00017, and Bb-00021, which were only detected in samples from heifers on this diet. In contrast, only three of the abundant rumen OTUs were more highly represented in SBGrass samples (*p* < 0.001). Similarly, 12 rumen OTUs were found in higher abundance in BCGrain heifers (*p* < 0.001); notably, this group also included the abovementioned Bb-00002, Bb-00017, and Bb-00021, which were similarly not detected in BCGrass samples (Table 4). Six other OTUs from the Blue Creek ranch were more abundant in the rumen of BCGrass heifers than in the BCGrain group (*p* < 0.001). Of these 20 most abundant rumen OTUs, only Bb-00003 and Bb-00021 showed nucleotide sequence identity levels of at least 97% with their closest valid relatives (*Prevotella ruminicola* and *Succinivibrio dextrinosolvens*, respectively). In contrast, the remaining OTUs had sequence identities to their respective closest bacterial species match that were lower than 93% (Table 4).

**Table 4:** Most abundant OTUs identified in the rumen of heifers from two ranches that transitioned from pasture to a free-choice grain diet.

**OTU**	**Mean relative abundance (%)**				***p-*value^#^**	**Closest valid taxon (%Id)**
	**SBGrass**	**SBGrain**	**BCGrass**	**BCGrain**		
SD_Bb-00002	0^a*^	1.94^b^	0^c*^	4.83^d^	< 0.001	*Ruminococcus bromii* (91.2%)
SD_Bb-00001	0.07^a^	2.42^b^	0.08^a^	4.12^ab^	< 0.001	*Mediterranea massiliensis* (83.7%)
SD_Bb-00006	0.40^a^	2.95^b^	0.27^a^	2.16^b^	< 0.001	*Saccharofermentans acetigenes* (87%)
SD_Bb-00004	0.07^a^	3.10^b^	0.04^a^	2.18^b^	< 0.001	*Negativibacillus massiliensis* (86.9%)
SD_Bb-00003	0.44^a^	4.43^b^	0.27^c^	0.15^d^	< 0.001	*Prevotella ruminicola* (97.9%)
SD_Bb-00005	0.31^a^	4.44^b^	0.11^c^	< 0.01^d^	< 0.001	*Prevotella brevis* (89.9%)
SD_Bb-00007	< 0.01^a^	2.09^b^	< 0.01^a^	0.86^b^	< 0.001	*Ruminococcus bromii* (93.2%)
SD_Bb-00009	0.53^a^	0.04^b^	1.56^c^	0.63^a^	< 0.001	*Christensenella massiliensis* (85.5%)
SD_Bb-00008	0.12^a^	2.68^b^	0.01^c^	0.02^c^	< 0.001	*Alistipes finegoldii* (84%)
SD_Bb-00017	0^a*^	2.77^b^	0^c*^	0.02^d^	< 0.001	*Prevotella copri* (88.1%)
SD_Bb-00018	0.01^a^	1.75^b^	< 0.01^a^	0.89^b^	< 0.001	*Prevotella buccalis* (88.7%)
SD_Bb-00011	0.39^a^	< 0.01^b^	1.61^c^	0.41^a^	< 0.001	*Christensenella massiliensis* (84.8%)
SD_Bb-00016	< 0.01^a^	2.46^b^	< 0.01^c^	0.01^a^	< 0.001	*Ruminobacter amylophilus* (83.5%)
SD_Bb-00010	0.18^a^	1.89^a^	0.25^a^	0.02^b^	< 0.001	*Prevotella ruminicola* (93.7%)
SD_Bb-00015	0.24^a^	0.43^b^	0.08^c^	1.47^d^	< 0.001	*Oscillibacter valericigenes* (87.9%)
SD_Bb-00012	< 0.01^a^	0.19^b^	< 0.01^a^	1.98^c^	< 0.001	*Anaerocolumna xylanovorans* (89.7%)
SD_Bb-00013	0.35^a^	0.69^ac^	0.12^b^	1.02^c^	< 0.001	*Oscillibacter valericigenes* (87%)
SD_Bb-00021	0^a*^	2.17^b^	0^c*^	0.05^d^	< 0.001	*Succinivibrio dextrinosolvens* (97.1%)
SD_Bb-00019	0.24^a^	0.56^a^	0.35^a^	0.94^b^	< 0.001	*Saccharofermentans acetigenes* (87%)
SD_Bb-00014	1.00^a^	0.01^b^	0.80^a^	0.09^c^	< 0.001	*Rhodopirellula pilleata* (83.2%)

SBGrass, Standing Butte Grass; SBGrain, Standing Butte Grain; BCGrass, Blue Creek Grass; BCGrain, Blue Creek grain.

^#^All OTUs shown displayed statistically significant differences in abundance (p < 0.05) across all groups based on the Kruskal–Wallis sum-rank test. Different superscripts in the same row indicate that the groups were significantly different by the Wilcoxon pairwise test.

^*^No reads were detected in any of the samples for this group. p-values were adjusted using the BH adjustment method.

%Id, % identity.

### OTU composition analysis of fecal bacterial communities

From a total of 36,418 OTUs identified from fecal samples in this portion of the study (Supplementary Table 2), 1.5% were predicted to correspond to known or already characterized bacterial species. In samples from the Standing Butte ranch, 16 of the 20 most abundant fecal OTUs were more highly represented in SBGrain samples, compared to four OTUs that were in higher abundance in SBGrass (*p* < 0.05; Table 5). Similarly, five fecal OTUs were found at higher levels in BCGrass, whereas 14 OTUs were more abundant in the BCGrain group (Table 5); notably, Bb-00020 represented 42.37% of all sequences analyzed from BCGrain. Similar to what was observed for rumen samples, three OTUs (Bb-00731, Bb-00740, and Bb-00746) were detected only in the bison that were fed a grain-based diet in both locations (Table 5). Of the 20 most abundant fecal OTUs identified, 10 had DNA sequence identities equal to or higher than 97% when compared to their respective closest known taxon (Table 5).

**Table 5:** Most abundant fecal OTUs identified in heifers from two ranches that transitioned from pasture to a free-choice grain diet.

**OTU**	**Mean relative abundance (%)**				***p-*value^#^**	**Closest valid taxon (%Id)**
	**SBGrass**	**SBGrain**	**BCGrass**	**BCGrain**		
SD_Bb-00020	0.07^a^	6.93^b^	0.13^c^	42.37^d^	< 0.001	*Succinivibrio dextrinosolvens* (97.1%)
SD_Bb-00727	< 0.01^a^	11.72^b^	< 0.01^a^	3.28^c^	< 0.001	*Faecalibacterium prausnitzii* (98.3%)
SD_Bb-00728	5.46^a^	< 0.01^b^	3.44^c^	< 0.01^b^	< 0.001	*Akkermansia muciniphila* (89.9%)
SD_Bb-00733	3.39^a^	< 0.01^b^	1.69^c^	< 0.01^b^	< 0.001	*Phocaeicola plebeius* (90.4%)
SD_Bb-00730	0.01^a^	1.09^b^	0.01^a^	4.18^c^	< 0.001	*Streptococcus lutetiensis* (95.8%)
SD_Bb-00729	< 0.01^a^	3.83^b^	< 0.01^a^	0.74^c^	< 0.001	*Eubacterium uniforme* (92.8%)
SD_Bb-00732	< 0.01^a^	3.27^b^	< 0.01^a^	0.86^c^	< 0.001	*Faecalibacterium prausnitzii* (93.9%)
SD_Bb-00731	0^a*^	3.69^b^	0^c*^	0.31^d^	< 0.001	*Prevotella stercorea* (98.8%)
SD_Bb-00734	< 0.01^a^	2.75^b^	< 0.01^a^	0.88^c^	< 0.001	*Prevotella copri* (97.2%)
SD_Bb-00735	< 0.01^a^	2.01^b^	< 0.01^a^	1.59^b^	< 0.001	*Blautia faecicola* (97%)
SD_Bb-00739	1.10^a^	0.04^b^	1.95^c^	< 0.01^d^	< 0.001	*Sporobacter termitidis* (88.2%)
SD_Bb-00399	< 0.01^a^	0.36^b^	0.02^c^	2.53^d^	< 0.001	*Lactobacillus amylovorus* (99.6%)
SD_Bb-00736	< 0.01^a^	2.16^b^	< 0.01^a^	0.67^c^	< 0.001	*Eubacterium rectale* (99.6%)
SD_Bb-00737	< 0.01^a^	1.77^b^	< 0.01^a^	0.97^b^	< 0.001	*Blautia luti* (97.8%)
SD_Bb-00004	0.21^a^	1.12^b^	0.43^c^	0.59^c^	< 0.001	*Negativibacillus massiliensis* (86.9%)
SD_Bb-00738	< 0.01^a^	1.22^b^	< 0.01^a^	1.12^b^	< 0.001	*Coprococcus catus* (96.7%)
SD_Bb-00741	0.21^a^	1.04^b^	0.82^b^	0.13^c^	< 0.001	*Romboutsia timonensis* (98.7%)
SD_Bb-00742	0.77^a^	0.37^b^	0.90^a^	0.01^c^	< 0.001	*Roseburia inulinivorans* (95.1%)
SD_Bb-00740	0^a*^	1.21^b^	0^c*^	0.93^b^	< 0.001	*Ruminococcus faecis* (100%)
SD_Bb-00746	0^a*^	0.72^b^	0^c*^	1.11^b^	< 0.001	*Sporobacter termitidis* (88.4%)

SBGrass, Standing Butte grass; SBGrain, Standing Butte grain; BCGrass, Blue Creek grass; BCGrain, Blue Creek grain.

^#^All OTUs shown displayed statistically significant differences in abundance (p < 0.05) across all groups based on the Kruskal–Wallis sum-rank test. Different superscripts in the same row indicate that groups were significantly different by the Wilcoxon pairwise test.

^*^No reads were detected in any of the samples for this group. p-values were adjusted using the BH adjustment method.

%Id, % identity.

## Discussion

Interest in bison studies stems from both a basic research perspective, as the largest wild ruminant in North America that has evolved to thrive in a variety of habitats, and a livestock production perspective, as understanding the fundamental physiology of bison has become essential to help guide management and husbandry decisions for North America's growing bison agricultural enterprise. This study thus aimed to achieve a better understanding of the bison gut microbial community and the effects of diet composition on the gut microbiome of bison, a ruminant host whose symbiotic communities are largely unexplored. Diet is well-known to have a major impact on gut microbiota (Petri et al., 2013; Newbold and Ramos-Morales, 2020), with the gastrointestinal tract environment adjusting swiftly to short-term diet changes (Clemmons et al., 2019). The focus of the present study was on bacterial communities because gut bacteria as a group are found at the highest cell densities, they represent the most genetically diverse group of symbionts, and they have the highest metabolic potential among the different types of symbiotic microorganisms (Wallace, 2008; McSweeney and Mackie, 2012). Comparative analyses of OTU composition, either by PCoA or on the most abundant individual OTUs, confirmed the dramatic impact of diet on the composition of both rumen and fecal bacterial communities in bison. Indeed, feeding a grain-based diet not only resulted in lower bacterial diversity but also in a dramatic change in OTU composition. The bison heifers on trial consumed, on average, ~50% of grain as part of their daily intake when on the grain-based diet, which would be less than what would be typically the inclusion level in the first step in adapting pasture-grazing cattle to a feedlot ration by producers in the U.S.A. While the impacts of feeding grain on the health of bison remain to be further investigated, the results from this study indicate that this level of grain intake was sufficient to cause dramatic changes in ruminal and fecal bacterial composition, which seemingly could not be mitigated by an equal intake of roughage. Feeding on ingredients such as corn and alfalfa, which have chemical characteristics that are different from the grasses and sedges that were grazed on pastures, combined with the likely variable intake of individual diet components from the grain-based diet by each heifer, provides a reasonable explanation for both reduced alpha diversity index values and differences in bacterial composition.

The majority of OTUs identified in this study were phylogenetically too distant from their closest relatives to reliably infer their function based on only 16S rRNA gene sequence comparisons. These unknown OTUs, which are likely to correspond to uncharacterized bacterial species, were more prevalent in samples collected during the grass-feeding phase and were also more common in the rumen than in fecal communities. For example, bison on a grain-based diet had fewer observed OTUs and fewer distinct types of bacteria than pasture-grazed bison. Furthermore, the rumen of the grass-fed bison displayed a high number of OTUs with lower abundances relative to the grain-fed bison, indicating a more diversified microbial environment.

Since members of the same taxonomic group may share common biochemical functions as a result of being in the same phylogenetic lineage, the main bison OTUs identified in this study were categorized based on their taxonomic affiliations as a strategy to gain further insight. Among Firmicutes, Ruminococcaceae were one of the predominant bacterial groups in both the rumen and fecal communities of bison across diets and locations. Isolates affiliated with this family have been characterized from the gut of various host animals and humans. They include species that primarily metabolize cellulose (Biddle et al., 2013), such as *Ruminococcus albus, Ruminococcus flavefaciens*, and *Ruminococcus champanellensis*, as well as others that can metabolize starch as their main substrate (Leitch et al., 2007), such as *R. bovis* and *R. bromii*. Such contrasting capabilities among different species of the same family may help explain why Ruminococcaceae were more highly represented in the rumen of heifers fed a grain-based diet while they were more abundant in the fecal communities of heifers on pasture. When considering the most abundant OTUs from both rumen and fecal communities, 10 of the OTUs affiliated with this family were more highly represented in the grain-based group from at least one location (Bb-00002, Bb-00004, Bb-00006, Bb-00007, Bb-00013, Bb-00015, Bb-00019, Bb-00727, Bb-00732, and Bb-00746), while Bb-00011 and Bb-00739 were the only Ruminococcaceae OTUs that were more highly represented in pasture-grazed heifers. Notably, Bb-00727 was the only OTU in this group with a high enough sequence identity to its closest match, *Faecalibacterium prausnitzii*, to allow reliable predictions of its potential functions in the gut. As *F. prausnitzii* is a known producer of butyrate, a short-chain fatty acid that is a preferred source of carbon and energy for colonic enterocytes (Salvi and Cowles, 2021), Bb-00727 would be deemed a favorable symbiont in the hindgut of bison by contributing to their host's gut health (Clausen and Mortensen, 1995). While we could not reliably predict the metabolic capabilities of the other Ruminococcaceae-affiliated OTUs because of their limited identity to their respective closest valid relatives (< 93.2%), their function may be inferred based on their composition patterns across dietary treatments.

Other well-represented Firmicutes families also showed distinctive composition patterns. Lachnospiraceae were found at higher levels in fecal samples, whereas in rumen samples, this family exhibited contrasting abundance patterns in response to grain-based diets between the two locations. This difference in representation could be either attributed to the unique metabolic capabilities of individual species and their respective ecological interactions within the rumen or to the influence of specific environmental conditions, including soil mineral and water composition, temperature, or other abiotic factors, which may vary between the two ranches. Of the seven most abundant fecal OTUs affiliated with this group, six were more highly represented in samples from the heifers that were fed the grain-based diet. Among these, Bb-00735, Bb-00736, and Bb-00737 were closely related to known bacterial species. *Blautia faecicola* (Bb-00735) was first isolated from human feces and is indicative of a healthy or well-functioning digestive system (Kim et al., 2020). The predominant fermentation end product produced by *B. faecicola* is acetate, which, as a major energy contributor to the host, can also be a major source of acetyl-CoA for lipid synthesis (Bergman, 1990). *Eubacterium rectale* (Bb-00736), a known contributor to healthy human gut environments (Martín et al., 2017; Karcher et al., 2020), can metabolize acetate to produce butyrate, a crucial SCFA for maintaining colonic health in humans and animals (Chun et al., 2017). *Blautia luti* (Bb-00737) is one of the dominant *Blautia* species of the human gut (Liu et al., 2021), contributing to the reduction of inflammation, a condition associated with metabolic disorders. To our knowledge, *B. luti* has not been isolated from the gastrointestinal tract of ruminants, but its presence in the hindgut of other mammals would be expected to benefit gut health in these other host species. Fecal communities of heifers on the grain-based diet also had a high abundance of Streptococcaceae, with one OTU, Bb-00020, representing 91.2 and 92.0% of all Streptococcaceae sequences in the Blue Creek and Standing Butte sets of samples, respectively. The Bb-00020 nucleotide sequence was found to be a perfect match to the 16S rRNA gene of *Streptococcus lutetiensis*, a bacterial species whose function remains to be determined in the ruminant gut. Note that it has been linked to respiratory diseases leading to sudden death in calves and may thus represent a potential pathogen (Clarke et al., 2016). In addition, unlike many members of the genus *Streptococcus, S. lutetiensis* does not appear to be associated with starch fermentation; rather, it has been reported to have coding sequences for beta-glucosidase activity, indicating the potential for cellulose hydrolysis (Schlegel et al., 2000; Poyart et al., 2002), which may suggest the presence of a novel strain of *S. lutetiensis* in the gut of bison with potential genes encoding for starch fermentation.

Consistent with a previous report on gut bacterial communities from bison (Bergmann, 2017) as well as with other studies on ruminants (Tapio et al., 2017; Furman et al., 2020; Huang et al., 2021; Zhang et al., 2021), Prevotellaceae represented the most abundant family-level bacterial group across rumen samples. Accordingly, five of the most abundant rumen OTUs were found to be affiliated with this family of Bacteroidetes, in comparison to only two abundant OTUs in fecal samples. As a group, members of the Prevotellaceae family are capable of a wide range of metabolic capabilities that contribute to rumen function, including the ability to the metabolize xylan (Miyazaki et al., 1997) and other complex carbohydrates (Flint et al., 2012), as well as proteins and peptides (Wallace et al., 1997). This may explain why dietary treatments did not appear to have as much of an impact on levels of Prevotellaceae in the rumen of bison heifers compared to other bacterial groups. Three of the abundant Prevotellaceae-affiliated OTUs (Bb-00003, Bb-00731, and Bb-00734) showed sufficiently high nucleotide sequence identity to *P. ruminicola, Prevotella stercorea*, and *Prevotella copri*, respectively. *Prevotella ruminicola* (Bb-00003) is a known starch utilizer and a well-characterized resident of the rumen that has been identified in several different hosts, where it can populate a variety of environmental niches (Avguštin et al., 1997; Ramšak et al., 2000; Purushe et al., 2010). *Prevotella stercorea* (SD_Bb-00731), which has been associated with plant-based diets in humans, does not appear to have been reported in the gut of ruminants so far (Precup and Vodnar, 2019). Since the main metabolic end products of *P. stercorea* include succinate (Hayashi et al., 2007), this OTU may be beneficial as a source of precursors for generating propionate, which is a preferred substrate for gluconeogenesis in ruminants (Hernandez-Sanabria et al., 2012). *Prevotella copri* (SD Bb-00734) has been found to possess genes encoding xylan hydrolyzing enzymes in its genome (Linares-Pastén et al., 2021), a favorable attribute as a symbiont since ruminants lack host-encoded digestive enzymes that can effectively contribute to the utilization of complex dietary fibers. Although additional research is needed to test this hypothesis, the abundance of *P. copri* in the gut of bison could explain their ability to perform better under poor-quality grasses, as this bacterial species has previously been identified as a potential marker for feed efficiency in beef cattle (Brooke et al., 2019).

Certain bacterial groups in the bison gastrointestinal tract that were affiliated with other phyla were also impacted by diet. Akkermansiaceae, a family belonging to Verrucomicrobia, were well-represented in fecal communities of pasture-grazed heifers from both ranches (Standing Butte: 6.38%; Blue Creek: 4.63%) but were at levels below detection in fecal samples from the heifers that were fed a grain-based diet. The predominant OTU affiliated with this group, Bb-00728 (Standing Butte: 5.46%; Blue Creek: 3.44%), was distantly related to its closest valid match, *Akkermansia muciniphila* (89.9%). While acknowledging the limited nucleotide sequence identity of Bb-00728 with this species, it is worth noting that *A. muciniphila* is a common resident of the hindgut, where it is involved in the breakdown of mucin (Hagi and Belzer, 2021). In most animals, mucin is produced by epithelial cells to form a protective layer that prevents colonization by pathogens and subsequent infections (Linden et al., 2008; Van Herreweghen et al., 2018). While excessive mucin degradation can disrupt host mucosal surfaces, resulting in undesired interactions of epithelial cells with pathogens (Derrien et al., 2010), controlled mucin breakdown by beneficial symbionts can have a positive effect on gut health by providing substrates, such as monosaccharides released from glycoproteins, to other beneficial bacteria that produce short-chain fatty acids such as acetate, propionate, and lactate (Van Herreweghen et al., 2018).

Most sequences affiliated with Proteobacteria belonged to Bb-00021, an OTU that was most prominently found in rumen and fecal samples from Standing Butte heifers fed a grain-based diet. Accordingly, this OTU was most closely related to *S. dextrinosolvens*, a bacterial species linked with high starch diets (Bryant, 1959; Indugu et al., 2017). *Succinivibrio* species have been positively associated with feed efficiency and animal performance; for instance, they were found to be four times higher in the rumen of low-methane-producing cattle compared to high-methane-producing cattle (Wallace et al., 2015). In addition to decreased methane emissions (Danielsson et al., 2017), positive correlations between *S. dextrinosolvens* and milk production (Indugu et al., 2017), fat milk content (Xue et al., 2018), and nitrogen utilization (Gomez-Alarcon et al., 1982; Hailemariam et al., 2020) have been reported. Since succinate, a precursor to propionate, is the main end product of *S. dextrinosolvens*, strains of this species may compete with methanogens by acting as an alternative hydrogen sink.

The contributions of microbial communities populating distal segments of the gut to the nutrition of their host are typically minor compared to the rumen. Instead, their primary role is maintaining gut health (O'Hara et al., 2020; Sanz-Fernandez et al., 2020). Considering the differences in chemical and biological conditions between the rumen and distal segments of the gut (Immig, 1996; O'Hara et al., 2020), it was not surprising to observe that each compartment harbored distinct microbial communities. For instance, only 791 OTUs (0.9% of rumen OTUs and 2.2% of fecal OTUs) were found to be shared between the rumen and fecal samples in this study. Each type of microbial community provides metabolic activities best suited for its environment, allowing them to perform their respective biological functions.

## Conclusion

Since gut microbial communities are dependent on the feed ingested by their host for energy and nutrients, diet is one of the main factors affecting the composition of gut microbial communities, as substrate availability determines the composition of the consortia of specialists that can prosper in a given segment or compartment of the gut. Owing to their limited sequence identity to known or validly characterized bacterial species, it has been a challenge to infer the function of most rumen OTUs identified in this study. However, as more unknown OTUs were found during the grass-feeding phase, these could potentially contribute to the ability of bison to perform better than cattle when consuming low-quality forage.

Further research in microbiome research aimed at elucidating the metabolic capabilities and potential functions of these bacterial species in the bison rumen would be beneficial. This research could help in the development of strategies to improve bison health and performance. Additionally, the findings could have broader implications for the production of traditional ruminant species, leading to the development of applications that can be beneficial to their overall production processes. Intriguingly, certain OTUs from bison fecal communities were predicted to be strains of beneficial bacterial species that are typical of human gut microbial environments rather than residents of the ruminant hindgut. Considering that these were identified in fecal samples from the heifers on the grain-based diet, it remains to be determined whether their presence is also beneficial for bison or if it is indicative of an imbalance resulting from feeding on a diet that bison did not evolve to utilize.

## Data availability statement

The datasets presented in this study can be found in online repositories under Bioproject PRJNA931600, available from the NCBI Sequence Read Archive at https://www.ncbi.nlm.nih.gov/sra.

## Ethics statement

The animal study was reviewed and approved by the South Dakota State University Institutional Animal Care and Use Committee.

## Author contributions

Conceptualization, study design, and methodology: BS-P and CK. Experimental work: JG and AF. Data analysis and data curation: AF and BS-P. Writing—original draft preparation: AF. Writing—review and editing: CK, JG, and BS-P. All authors have read and agreed to the published version of the manuscript.
