# Supplementary material for: Effects of grain-based diets on the rumen and fecal bacterial communities of the North American bison (Bison bison)
Source: Front Microbiol. 2023 Jul 6;14:1163423. doi: 10.3389/fmicb.2023.1163423 (PMC10359189; doi:10.3389/fmicb.2023.1163423)
Supplement: Supplementary file 1 [file Image_1.PDF]

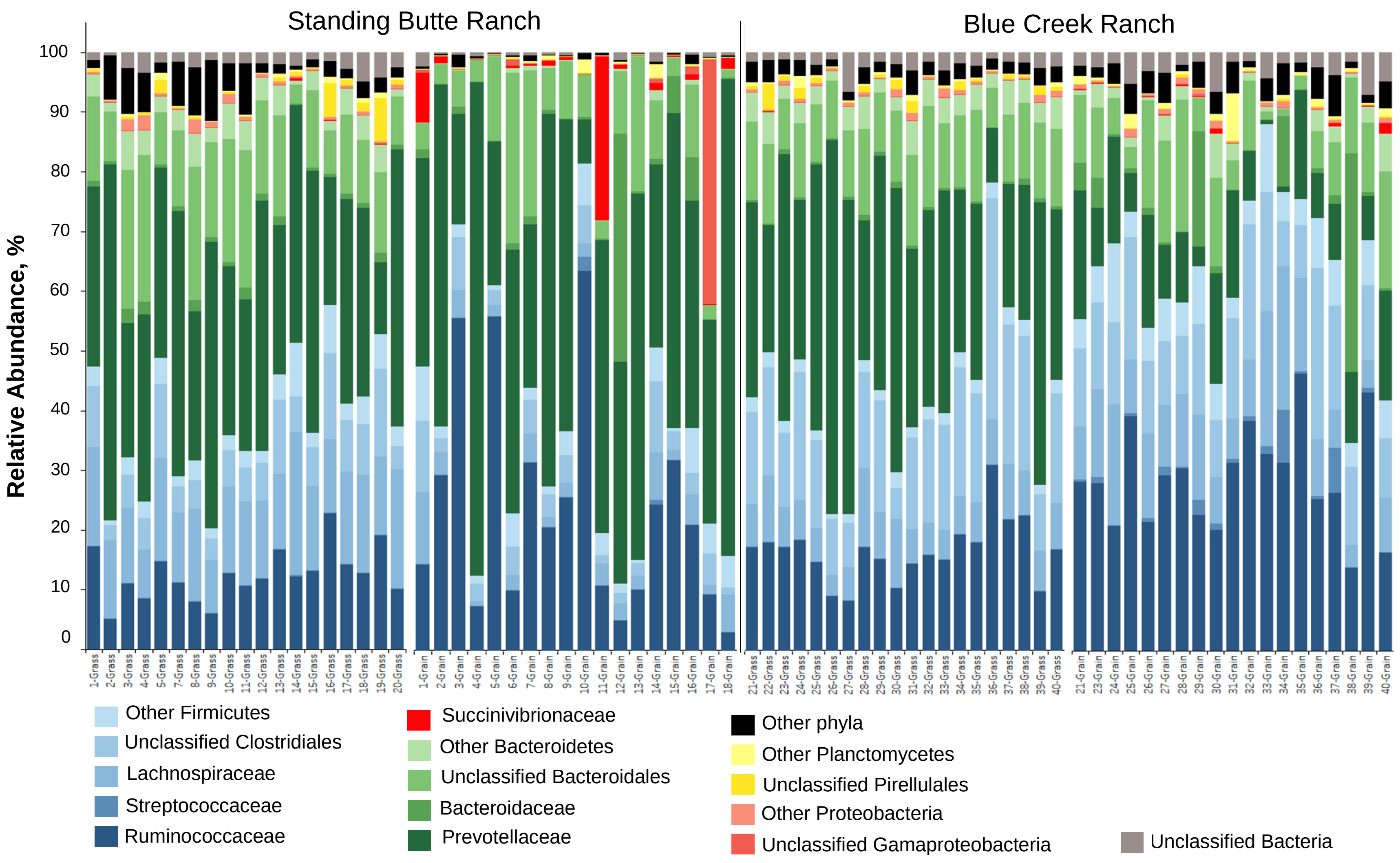

Standing Butte Ranch
Blue Creek Ranch
Relative Abundance, %
100
90
80
70
60
50
40
30
20
10
0
Other Firmicutes
Unclassified Clostridiales
Lachnospiraceae
Streptococcaceae
Ruminococcaceae
Succinivibrionaceae
Other Bacteroidetes
Unclassified Bacteroidales
Bacteroidaceae
Prevotellaceae
Other phyla
Other Planctomycetes
Unclassified Pirellulales
Other Proteobacteria
Unclassified Gamaproteobacteria
Unclassified Bacteria

Supplementary Figure 1. ***Phylum and family level taxonomic composition of rumen bacterial communities in individual bison heifers from two separate ranches that transitioned from grazing on pasture to a grain-based free choice diet.*** Families belonging to the same phylum are represented by different shades of the same color: Bacteroidetes (green), Firmicutes (blue), Proteobacteria (Red) and Planctomycetes (Yellow).
