# Supplementary material for: Effects of grain-based diets on the rumen and fecal bacterial communities of the North American bison (Bison bison)
Source: Front Microbiol. 2023 Jul 6;14:1163423. doi: 10.3389/fmicb.2023.1163423 (PMC10359189; doi:10.3389/fmicb.2023.1163423)
Supplement: Supplementary file 2 [file Image_2.PDF]

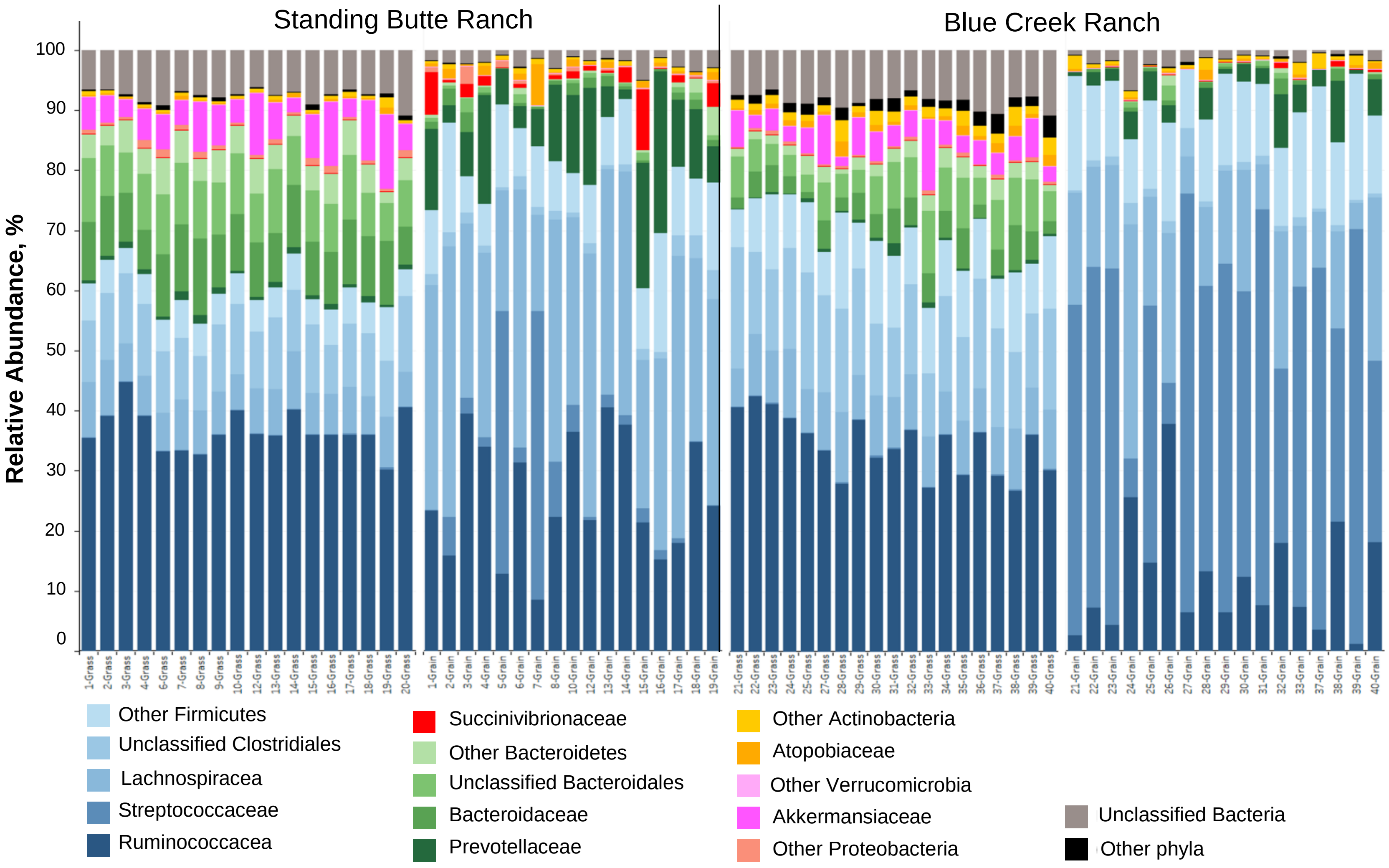

Supplementary Figure 2. ***Phylum and family level taxonomic composition of fecal bacterial communities in individual bison heifers from two separate ranches that transitioned from grazing on pasture to a grain-based free choice diet***. Families belonging to the same phylum are represented by different shades of the same color: Bacteroidetes (green), Firmicutes (blue), Actinobacteria (Orange) and Verrucomicrobia (Pink).
