# Supplementary material for: Effects of grain-based diets on the rumen and fecal bacterial communities of the North American bison (Bison bison)
Source: Front Microbiol. 2023 Jul 6;14:1163423. doi: 10.3389/fmicb.2023.1163423 (PMC10359189; doi:10.3389/fmicb.2023.1163423)
Supplement: Supplementary file 3 [file Table_1.DOCX]

| **Supplementary Table 1.** Main native grasses populating the pastures of the Blue Creek ranch and Standing Butte ranch. | |
| --- | --- |
| **Standing Butte ranch** | **Blue Creek ranch** |
| **Native grasses**   - Western Wheatgrass - Slender Wheatgrass - Needle and thread - Green needle grass - Porcupine grass - Side oats grama - Little bluestem - Buffalo grass - Blue grama - Prairie June grass - Plains muhly   **Native forbs**     - Western Yarrow - Wild onion - Ragweed - Milkvetch - Wavey-leaf thistle - Prairie clover - Scurf-pea - Coneflower - Goldenrod. | **Native grasses**   - Sand Bluestem - Prairie sandreed - Needle and thread - Blue grama - Sand love-grass - Switchgrass - Little bluestem - Sedges - Prairie June grass - Sand dropseed   **Native forbs**   - Spiderwort - Ragweed - Scurf-pea - Goldenrod - Milk vetch - Sunflower |
